# (*E*)-Ethyl 3-(3-bromo­phen­yl)-2-cyano­acrylate

**DOI:** 10.1107/S1600536809039518

**Published:** 2009-10-03

**Authors:** Yin Ye, Wei-Li Shen, Xian-Wen Wei

**Affiliations:** a College of Chemistry and Materials Science, Anhui Key Laboratory of Functional Molecular Solid, Anhui Normal University, Wuhu 241000, People’s Republic of China

## Abstract

The title mol­ecule, C_12_H_10_BrNO_2_, adopts an *E* configuration with respect to the C=C bond of the acrylate unit. In the crystal structure, mol­ecules are connected by a pair of inter­molecular C—H⋯O hydrogen bonds, forming a centrosymmetric dimer.

## Related literature

For the synthesis, see: Lapworth & Baker (1927 ▶). For the title compound as an inter­mediate in drug synthesis, see: Obniska *et al.* (2005 ▶).
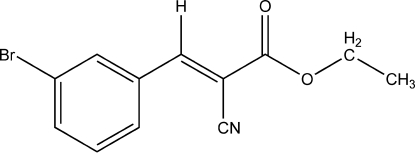


## Experimental

### 

#### Crystal data

C_12_H_10_BrNO_2_
*M* *_r_* = 280.12
Monoclinic, 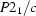

*a* = 7.6147 (7) Å
*b* = 21.6015 (19) Å
*c* = 7.6044 (7) Å
β = 110.370 (1)°
*V* = 1172.62 (18) Å^3^
*Z* = 4
Mo *K*α radiation
μ = 3.49 mm^−1^
*T* = 298 K
0.17 × 0.14 × 0.09 mm

#### Data collection

Bruker SMART APEX CCD area-detector diffractometer
Absorption correction: multi-scan (**SADABS**; Sheldrick, 1996 ▶) *T*_min_ = 0.597, *T*_max_ = 0.751
10143 measured reflections
2698 independent reflections
1730 reflections with *I* > 2σ(*I*)
*R*_int_ = 0.038

#### Refinement

*R*[*F* ^2^ > 2σ(*F* ^2^)] = 0.034
*wR*(*F* ^2^) = 0.081
*S* = 1.00
2698 reflections
146 parameters
H-atom parameters constrained
Δρ_max_ = 0.21 e Å^−3^
Δρ_min_ = −0.33 e Å^−3^

### 

Data collection: *SMART* (Bruker, 2000 ▶); cell refinement: *SAINT* (Bruker, 2000 ▶); data reduction: *SAINT*; program(s) used to solve structure: *SHELXS97* (Sheldrick, 2008 ▶); program(s) used to refine structure: *SHELXL97* (Sheldrick, 2008 ▶); molecular graphics: *SHELXTL* (Sheldrick, 2008 ▶); software used to prepare material for publication: *SHELXTL*.

## Supplementary Material

Crystal structure: contains datablocks I, global. DOI: 10.1107/S1600536809039518/is2461sup1.cif

Structure factors: contains datablocks I. DOI: 10.1107/S1600536809039518/is2461Isup2.hkl

Additional supplementary materials:  crystallographic information; 3D view; checkCIF report

## Figures and Tables

**Table 1:** Hydrogen-bond geometry (Å, °)

*D*—H⋯*A*	*D*—H	H⋯*A*	*D*⋯*A*	*D*—H⋯*A*
C6—H6⋯O1^i^	0.93	2.47	3.323 (3)	152

Symmetry code: (i) 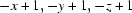.

**Fig. 1.**
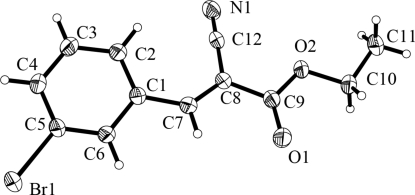
The molecular structure of (I), showing the atom-labelling scheme. Displacement ellipsoids are drawn at the 30% probability level.

**Fig. 2.**
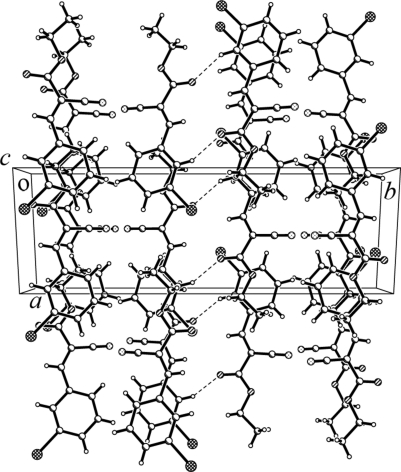
A packing diagram of (I) viewed down the c axis. Dotted lines show the C—H···O hydrogen bonds.

## supplementary crystallographic information

### Comment

The title compound is an important intermediate in drugs synthesis (Obniska *et al.*, 2005). In this paper, we report the structure of the title compound (I). In (I), the molecule adopts an *E* configuration. Both the C7═C8 and the cyano (C12≡N1) groups deviate from the mean plane of the benzene C1–C6 ring. The crystal packing is stabilized by intermolecular non-classic C—H···O hydrogen bonds.

### Experimental

The compound (I) was obtained by reaction of 3-bromobenzaldehyde (36.8 g, 0.2 mol) and ethyl 2-cyanoacetate (22.6 g, 0.2 mol) in the absolute ethanol (180 ml) containing 3.2 ml triethylamine according to the reported method (Lapworth *et al.*, 1927). Single crystals suitable for X-ray diffraction were obtained by evaporation of an ethanol solution at room temperature.

### Refinement

H atoms bonded to C atoms were introduced at calculated positions (C—H = 0.93–0.97 Å) and refined using a riding model, with *U*_iso_(H) = 1.2*U*_eq_(C).

### Crystal data

**Table:**

C_12_H_10_BrNO_2_	*F*(000) = 560
*M**_r_* = 280.12	*D*_x_ = 1.587 Mg m^−^^3^
Monoclinic, *P*2_1_/*c*	Mo *K*α radiation, λ = 0.71073 Å
Hall symbol: -P 2ybc	Cell parameters from 2803 reflections
*a* = 7.6147 (7) Å	θ = 2.9–25.8°
*b* = 21.6015 (19) Å	µ = 3.49 mm^−^^1^
*c* = 7.6044 (7) Å	*T* = 298 K
β = 110.370 (1)°	Block, colorless
*V* = 1172.62 (18) Å^3^	0.17 × 0.14 × 0.09 mm
*Z* = 4	

### Data collection

**Table:**

Bruker SMART APEX CCD area-detector diffractometer	2698 independent reflections
Radiation source: fine-focus sealed tube	1730 reflections with *I* > 2σ(*I*)
graphite	*R*_int_ = 0.038
φ and ω scans	θ_max_ = 27.6°, θ_min_ = 1.9°
Absorption correction: multi-scan (*SADABS*; Sheldrick, 1996)	*h* = −9→9
*T*_min_ = 0.597, *T*_max_ = 0.751	*k* = −28→28
10143 measured reflections	*l* = −8→9

### Refinement

**Table:**

Refinement on *F*^2^	Primary atom site location: structure-invariant direct methods
Least-squares matrix: full	Secondary atom site location: difference Fourier map
*R*[*F*^2^ > 2σ(*F*^2^)] = 0.034	Hydrogen site location: inferred from neighbouring sites
*wR*(*F*^2^) = 0.081	H-atom parameters constrained
*S* = 1.00	*w* = 1/[σ^2^(*F*_o_^2^) + (0.0337*P*)^2^ + 0.2149*P*] where *P* = (*F*_o_^2^ + 2*F*_c_^2^)/3
2698 reflections	(Δ/σ)_max_ = 0.001
146 parameters	Δρ_max_ = 0.21 e Å^−^^3^
0 restraints	Δρ_min_ = −0.33 e Å^−^^3^

### Special details

**Table:**

Geometry. All e.s.d.'s (except the e.s.d. in the dihedral angle between two l.s. planes) are estimated using the full covariance matrix. The cell e.s.d.'s are taken into account individually in the estimation of e.s.d.'s in distances, angles and torsion angles; correlations between e.s.d.'s in cell parameters are only used when they are defined by crystal symmetry. An approximate (isotropic) treatment of cell e.s.d.'s is used for estimating e.s.d.'s involving l.s. planes.
Refinement. Refinement of *F*^2^ against ALL reflections. The weighted *R*-factor *wR* and goodness of fit *S* are based on *F*^2^, conventional *R*-factors *R* are based on *F*, with *F* set to zero for negative *F*^2^. The threshold expression of *F*^2^ > σ(*F*^2^) is used only for calculating *R*-factors(gt) *etc*. and is not relevant to the choice of reflections for refinement. *R*-factors based on *F*^2^ are statistically about twice as large as those based on *F*, and *R*- factors based on ALL data will be even larger.

### Fractional atomic coordinates and isotropic or equivalent isotropic displacement parameters (Å^2^)

**Table:**

	*x*	*y*	*z*	*U*_iso_*/*U*_eq_	
Br1	1.32627 (4)	0.459872 (14)	0.80815 (5)	0.06743 (14)	
C5	1.1401 (4)	0.40161 (12)	0.6781 (4)	0.0491 (7)	
C6	0.9564 (4)	0.42010 (12)	0.6053 (4)	0.0464 (6)	
H6	0.9240	0.4608	0.6193	0.056*	
C7	0.6257 (4)	0.39971 (12)	0.4366 (4)	0.0481 (6)	
H7	0.6060	0.4373	0.4862	0.058*	
C1	0.8179 (3)	0.37725 (11)	0.5098 (4)	0.0460 (6)	
N1	0.4728 (3)	0.27353 (12)	0.1299 (4)	0.0694 (7)	
C8	0.4721 (3)	0.37490 (11)	0.3089 (3)	0.0438 (6)	
C4	1.1939 (4)	0.34169 (14)	0.6612 (4)	0.0626 (8)	
H4	1.3192	0.3301	0.7101	0.075*	
C2	0.8709 (4)	0.31640 (13)	0.4958 (4)	0.0629 (8)	
H2	0.7806	0.2870	0.4362	0.075*	
C10	−0.0327 (4)	0.40634 (13)	0.0878 (4)	0.0579 (8)	
H10A	−0.0360	0.4404	0.0035	0.069*	
H10B	−0.0578	0.4225	0.1957	0.069*	
C11	−0.1756 (4)	0.35851 (14)	−0.0101 (5)	0.0682 (9)	
H11A	−0.1452	0.3412	−0.1122	0.102*	
H11B	−0.2972	0.3774	−0.0577	0.102*	
H11C	−0.1759	0.3263	0.0768	0.102*	
C3	1.0573 (4)	0.29951 (14)	0.5701 (5)	0.0754 (10)	
H3	1.0912	0.2588	0.5582	0.090*	
O2	0.1493 (2)	0.37612 (8)	0.1469 (2)	0.0517 (5)	
O1	0.2787 (3)	0.46073 (9)	0.3104 (3)	0.0703 (6)	
C12	0.4724 (3)	0.31789 (13)	0.2110 (4)	0.0500 (7)	
C9	0.2926 (4)	0.40931 (13)	0.2581 (4)	0.0493 (7)	

### Atomic displacement parameters (Å^2^)

**Table:**

	*U*^11^	*U*^22^	*U*^33^	*U*^12^	*U*^13^	*U*^23^
Br1	0.04546 (19)	0.0595 (2)	0.0794 (2)	−0.00844 (14)	−0.00088 (15)	−0.00303 (16)
C5	0.0422 (15)	0.0497 (16)	0.0478 (16)	−0.0039 (12)	0.0061 (13)	0.0000 (12)
C6	0.0477 (16)	0.0381 (14)	0.0488 (16)	0.0000 (11)	0.0109 (12)	0.0000 (12)
C7	0.0445 (15)	0.0409 (14)	0.0548 (17)	−0.0001 (11)	0.0119 (13)	−0.0013 (12)
C1	0.0394 (14)	0.0436 (14)	0.0476 (16)	−0.0031 (11)	0.0058 (12)	0.0004 (12)
N1	0.0519 (15)	0.0609 (16)	0.092 (2)	−0.0110 (12)	0.0204 (14)	−0.0237 (15)
C8	0.0381 (14)	0.0438 (14)	0.0474 (16)	−0.0023 (11)	0.0123 (12)	−0.0003 (12)
C4	0.0421 (16)	0.0558 (17)	0.076 (2)	0.0073 (13)	0.0024 (15)	−0.0037 (15)
C2	0.0503 (17)	0.0469 (16)	0.074 (2)	−0.0019 (13)	−0.0010 (15)	−0.0078 (14)
C10	0.0349 (15)	0.0605 (18)	0.073 (2)	0.0057 (13)	0.0125 (14)	−0.0015 (15)
C11	0.0390 (16)	0.077 (2)	0.079 (2)	−0.0025 (15)	0.0092 (15)	0.0018 (17)
C3	0.0539 (19)	0.0490 (17)	0.102 (3)	0.0099 (14)	0.0000 (18)	−0.0102 (17)
O2	0.0350 (10)	0.0511 (11)	0.0634 (12)	−0.0005 (8)	0.0098 (9)	−0.0070 (9)
O1	0.0505 (12)	0.0600 (13)	0.0904 (16)	0.0028 (10)	0.0119 (11)	−0.0251 (11)
C12	0.0329 (14)	0.0529 (16)	0.0594 (18)	−0.0069 (12)	0.0098 (13)	−0.0009 (14)
C9	0.0410 (15)	0.0534 (17)	0.0505 (17)	−0.0041 (12)	0.0120 (13)	−0.0033 (13)

### Geometric parameters (Å, °)

**Table:**

Br1—C5	1.896 (2)	C4—H4	0.9300
C5—C6	1.372 (3)	C2—C3	1.382 (4)
C5—C4	1.377 (4)	C2—H2	0.9300
C6—C1	1.401 (3)	C10—O2	1.454 (3)
C6—H6	0.9300	C10—C11	1.497 (4)
C7—C8	1.344 (3)	C10—H10A	0.9700
C7—C1	1.456 (3)	C10—H10B	0.9700
C7—H7	0.9300	C11—H11A	0.9600
C1—C2	1.390 (4)	C11—H11B	0.9600
N1—C12	1.140 (3)	C11—H11C	0.9600
C8—C12	1.439 (4)	C3—H3	0.9300
C8—C9	1.484 (4)	O2—C9	1.333 (3)
C4—C3	1.374 (4)	O1—C9	1.197 (3)
			
C6—C5—C4	122.0 (2)	O2—C10—C11	107.1 (2)
C6—C5—Br1	119.3 (2)	O2—C10—H10A	110.3
C4—C5—Br1	118.7 (2)	C11—C10—H10A	110.3
C5—C6—C1	119.6 (2)	O2—C10—H10B	110.3
C5—C6—H6	120.2	C11—C10—H10B	110.3
C1—C6—H6	120.2	H10A—C10—H10B	108.6
C8—C7—C1	130.5 (2)	C10—C11—H11A	109.5
C8—C7—H7	114.8	C10—C11—H11B	109.5
C1—C7—H7	114.8	H11A—C11—H11B	109.5
C2—C1—C6	118.6 (2)	C10—C11—H11C	109.5
C2—C1—C7	124.5 (2)	H11A—C11—H11C	109.5
C6—C1—C7	116.9 (2)	H11B—C11—H11C	109.5
C7—C8—C12	123.9 (2)	C4—C3—C2	121.3 (3)
C7—C8—C9	118.6 (2)	C4—C3—H3	119.3
C12—C8—C9	117.5 (2)	C2—C3—H3	119.3
C3—C4—C5	118.2 (3)	C9—O2—C10	115.9 (2)
C3—C4—H4	120.9	N1—C12—C8	178.3 (3)
C5—C4—H4	120.9	O1—C9—O2	124.2 (3)
C3—C2—C1	120.2 (3)	O1—C9—C8	124.0 (2)
C3—C2—H2	119.9	O2—C9—C8	111.8 (2)
C1—C2—H2	119.9		
			
C4—C5—C6—C1	−0.3 (4)	C7—C1—C2—C3	−179.9 (3)
Br1—C5—C6—C1	−179.72 (19)	C5—C4—C3—C2	0.5 (5)
C5—C6—C1—C2	1.6 (4)	C1—C2—C3—C4	0.9 (5)
C5—C6—C1—C7	179.8 (2)	C11—C10—O2—C9	−171.7 (2)
C8—C7—C1—C2	−17.9 (5)	C10—O2—C9—O1	0.5 (4)
C8—C7—C1—C6	164.1 (3)	C10—O2—C9—C8	−179.3 (2)
C1—C7—C8—C12	−1.8 (5)	C7—C8—C9—O1	7.3 (4)
C1—C7—C8—C9	−178.8 (3)	C12—C8—C9—O1	−169.9 (3)
C6—C5—C4—C3	−0.8 (5)	C7—C8—C9—O2	−172.8 (2)
Br1—C5—C4—C3	178.7 (2)	C12—C8—C9—O2	9.9 (3)
C6—C1—C2—C3	−1.9 (5)		

### Hydrogen-bond geometry (Å, °)

**Table:**

*D*—H···*A*	*D*—H	H···*A*	*D*···*A*	*D*—H···*A*
C6—H6···O1^i^	0.93	2.47	3.323 (3)	152

Symmetry codes: (i) −*x*+1, −*y*+1, −*z*+1.
